# Supplementary material for: Detailed molecular and epigenetic characterization of the pig IPEC-J2 and chicken SL-29 cell lines
Source: iScience. 2023 Feb 20;26(3):106252. doi: 10.1016/j.isci.2023.106252 (PMC10018572; doi:10.1016/j.isci.2023.106252)
Supplement: Data S2. Complete homer output for identified motifs in Chicken SL-29, related to Tables 5 and 6 — Homer motif analysis results for histone modifications H3K4me1, H3K4me3, H3K27ac, enhancers, and ATAC-seq of chicken SL-29 cell line. Parameters for possible false positives is as mentioned earlier for S5. [file mmc3.zip › Data_S2/S6/Chicken_SL_29/peak_files_H3K4me1/knownResults.html]

peak\_files\_H3K4me1 - Homer Known Motif Enrichment Results


# Homer Known Motif Enrichment Results (peak\_files\_H3K4me1)

Homer *de novo* Motif Results  
Gene Ontology Enrichment Results  
Known Motif Enrichment Results (txt file)  
Total Target Sequences = 49829, Total Background Sequences = 49840

|  |  |  |  |  |  |  |  |  |  |  |  |
| --- | --- | --- | --- | --- | --- | --- | --- | --- | --- | --- | --- |
| Rank | Motif | Name | P-value | log P-pvalue | q-value (Benjamini) | # Target Sequences with Motif | % of Targets Sequences with Motif | # Background Sequences with Motif | % of Background Sequences with Motif | Motif File | SVG |
| 1 | C T A G T C G A A C G T A C T G C G T A T A G C C G A T G T A C C G T A A G C T G A T C G T A C | Jun-AP1(bZIP)/K562-cJun-ChIP-Seq(GSE31477)/Homer | 1e-76 | -1.758e+02 | 0.0000 | 1749.0 | 3.51% | 1091.5 | 2.19% | motif file (matrix) | svg |
| 2 | C T A G T C G A C G A T A C T G C G T A T A C G A G C T T G A C G C T A A C G T G A T C T A G C | Fosl2(bZIP)/3T3L1-Fosl2-ChIP-Seq(GSE56872)/Homer | 1e-71 | -1.654e+02 | 0.0000 | 2409.0 | 4.83% | 1644.1 | 3.30% | motif file (matrix) | svg |
| 3 | A C T G C T A G T C G A C G A T C A T G G C T A A T C G C G A T G T A C G C T A A G C T G T A C | Fra1(bZIP)/BT549-Fra1-ChIP-Seq(GSE46166)/Homer | 1e-66 | -1.531e+02 | 0.0000 | 4671.0 | 9.37% | 3629.0 | 7.27% | motif file (matrix) | svg |
| 4 | C A T G C T A G T C G A A C G T A C T G C G T A T A G C C G A T T G A C C G T A A G C T G A T C | Fra2(bZIP)/Striatum-Fra2-ChIP-Seq(GSE43429)/Homer | 1e-64 | -1.492e+02 | 0.0000 | 3757.0 | 7.53% | 2834.6 | 5.68% | motif file (matrix) | svg |
| 5 | C T A G T C G A A C G T A C T G C G T A A T G C A C G T G T A C C G T A A G C T G A T C G T A C | Atf3(bZIP)/GBM-ATF3-ChIP-Seq(GSE33912)/Homer | 1e-63 | -1.465e+02 | 0.0000 | 5492.0 | 11.01% | 4386.9 | 8.79% | motif file (matrix) | svg |
| 6 | C A G T T G C A A C G T A C T G C G T A A T C G C G A T T G A C C G T A A C G T | BATF(bZIP)/Th17-BATF-ChIP-Seq(GSE39756)/Homer | 1e-61 | -1.418e+02 | 0.0000 | 5187.0 | 10.40% | 4128.4 | 8.28% | motif file (matrix) | svg |
| 7 | A C T G C A T G T C G A A C G T A C T G C G T A A T C G A C G T G T A C C G T A G A C T A G T C | Fos(bZIP)/TSC-Fos-ChIP-Seq(GSE110950)/Homer | 1e-61 | -1.410e+02 | 0.0000 | 4900.0 | 9.82% | 3873.4 | 7.76% | motif file (matrix) | svg |
| 8 | C T A G T C G A G C A T C A T G G C T A T A G C C G A T G T A C C T G A A G C T | JunB(bZIP)/DendriticCells-Junb-ChIP-Seq(GSE36099)/Homer | 1e-60 | -1.393e+02 | 0.0000 | 4346.0 | 8.71% | 3384.4 | 6.78% | motif file (matrix) | svg |
| 9 | T C G A A C G T C A T G G C T A T A G C C G A T G T A C G C T A A C G T A T G C | AP-1(bZIP)/ThioMac-PU.1-ChIP-Seq(GSE21512)/Homer | 1e-52 | -1.209e+02 | 0.0000 | 5904.0 | 11.84% | 4861.0 | 9.74% | motif file (matrix) | svg |
| 10 | C G A T T A C G T G A C G A C T C A T G C G T A T A C G A C G T G T A C C T G A | Bach2(bZIP)/OCILy7-Bach2-ChIP-Seq(GSE44420)/Homer | 1e-30 | -6.973e+01 | 0.0000 | 1475.0 | 2.96% | 1080.7 | 2.17% | motif file (matrix) | svg |
| 11 | T G C A C T G A A G T C G T C A A C T G A C T G C G T A C G T A C T G A A G C T | EWS:FLI1-fusion(ETS)/SK\_N\_MC-EWS:FLI1-ChIP-Seq(SRA014231)/Homer | 1e-20 | -4.622e+01 | 0.0000 | 3696.0 | 7.41% | 3178.5 | 6.37% | motif file (matrix) | svg |
| 12 | A T G C A G T C C T G A A G T C C G A T A C G T A G T C A G T C A C G T A T C G G A C T A C G T | Etv2(ETS)/ES-ER71-ChIP-Seq(GSE59402)/Homer | 1e-19 | -4.593e+01 | 0.0000 | 6328.0 | 12.69% | 5663.5 | 11.35% | motif file (matrix) | svg |
| 13 | T A C G T C G A C A G T A C T G G C T A A T G C C G A T G T A C C G T A A C T G T A G C C G T A | NF-E2(bZIP)/K562-NFE2-ChIP-Seq(GSE31477)/Homer | 1e-19 | -4.451e+01 | 0.0000 | 508.0 | 1.02% | 330.7 | 0.66% | motif file (matrix) | svg |
| 14 | T C G A C T G A T A G C T G A C T C A G T C A G C G T A C G T A T C A G A G C T | ETV1(ETS)/GIST48-ETV1-ChIP-Seq(GSE22441)/Homer | 1e-17 | -3.937e+01 | 0.0000 | 8393.0 | 16.83% | 7699.2 | 15.43% | motif file (matrix) | svg |
| 15 | T C G A A G C T A C G T A C G T A G T C A G T C A C G T A T C G G A C T A T C G | EWS:ERG-fusion(ETS)/CADO\_ES1-EWS:ERG-ChIP-Seq(SRA014231)/Homer | 1e-16 | -3.879e+01 | 0.0000 | 6003.0 | 12.04% | 5408.4 | 10.84% | motif file (matrix) | svg |
| 16 | C G T A C G T A C G T A G C A T G C A T T A C G G T A C G A C T A C T G C G T A A T C G A C G T G T A C C G T A A G C T | Bach1(bZIP)/K562-Bach1-ChIP-Seq(GSE31477)/Homer | 1e-16 | -3.693e+01 | 0.0000 | 546.0 | 1.09% | 376.0 | 0.75% | motif file (matrix) | svg |
| 17 | T A G C T A G C G C A T C A T G A C T G G C T A C G T A A C G T A C T G G A T C | TEAD4(TEA)/Tropoblast-Tead4-ChIP-Seq(GSE37350)/Homer | 1e-14 | -3.376e+01 | 0.0000 | 6141.0 | 12.31% | 5582.0 | 11.19% | motif file (matrix) | svg |
| 18 | C T G A T A G C T G A C T C A G C T A G G T C A C G T A T C A G A G C T T C A G | ETV4(ETS)/HepG2-ETV4-ChIP-Seq(ENCODE)/Homer | 1e-14 | -3.365e+01 | 0.0000 | 5850.0 | 11.73% | 5304.3 | 10.63% | motif file (matrix) | svg |
| 19 | T C G A T A G C G T C A A C T G A C T G C G T A C G T A C T A G A G C T T C A G | ERG(ETS)/VCaP-ERG-ChIP-Seq(GSE14097)/Homer | 1e-13 | -3.191e+01 | 0.0000 | 10989.0 | 22.03% | 10298.3 | 20.64% | motif file (matrix) | svg |
| 20 | C G T A C G T A C G T A G C A T G C A T A C T G G T A C G A C T C T A G G C T A T A C G G A C T T G A C C G T A A G C T | NFE2L2(bZIP)/HepG2-NFE2L2-ChIP-Seq(Encode)/Homer | 1e-13 | -3.019e+01 | 0.0000 | 518.0 | 1.04% | 368.3 | 0.74% | motif file (matrix) | svg |
| 21 | T C A G T C A G T A G C A G T C C T G A A G T C C T A G A C G T A C T G A T C G | c-Myc(bHLH)/mES-cMyc-ChIP-Seq(GSE11431)/Homer | 1e-12 | -2.990e+01 | 0.0000 | 3085.0 | 6.19% | 2706.7 | 5.43% | motif file (matrix) | svg |
| 22 | G T C A G C A T A C T G G T A C G A C T A C T G G C T A A T C G C A G T G T A C C G T A A G C T | Nrf2(bZIP)/Lymphoblast-Nrf2-ChIP-Seq(GSE37589)/Homer | 1e-12 | -2.958e+01 | 0.0000 | 413.0 | 0.83% | 282.3 | 0.57% | motif file (matrix) | svg |
| 23 | C T G A T C A G G T A C G C T A A C T G T G A C G C A T C A T G | SCL(bHLH)/HPC7-Scl-ChIP-Seq(GSE13511)/Homer | 1e-11 | -2.702e+01 | 0.0000 | 35715.0 | 71.61% | 35017.9 | 70.19% | motif file (matrix) | svg |
| 24 | T A C G T C G A T A G C A G T C C G T A A G T C C T A G G C A T A C T G A T C G | n-Myc(bHLH)/mES-nMyc-ChIP-Seq(GSE11431)/Homer | 1e-11 | -2.620e+01 | 0.0000 | 4240.0 | 8.50% | 3828.9 | 7.68% | motif file (matrix) | svg |
| 25 | T G A C A G T C C T G A T G A C C G T A A C G T A C G T A G T C A G T C C G T A | TEAD1(TEAD)/HepG2-TEAD1-ChIP-Seq(Encode)/Homer | 1e-11 | -2.620e+01 | 0.0000 | 7240.0 | 14.52% | 6715.5 | 13.46% | motif file (matrix) | svg |
| 26 | A G T C C T G A A G T C C G A T C A G T G A T C A T G C A C T G A T C G G A C T | Fli1(ETS)/CD8-FLI-ChIP-Seq(GSE20898)/Homer | 1e-11 | -2.579e+01 | 0.0000 | 6420.0 | 12.87% | 5926.4 | 11.88% | motif file (matrix) | svg |
| 27 | G T A C G A C T C G T A C T G A T C G A C G T A G C T A C A G T C T G A T A C G | Mef2a(MADS)/HL1-Mef2a.biotin-ChIP-Seq(GSE21529)/Homer | 1e-11 | -2.558e+01 | 0.0000 | 3901.0 | 7.82% | 3510.9 | 7.04% | motif file (matrix) | svg |
| 28 | T C G A T A G C T G C A A C T G A C T G C G T A C G T A C T A G G A C T T A C G | ETS1(ETS)/Jurkat-ETS1-ChIP-Seq(GSE17954)/Homer | 1e-10 | -2.530e+01 | 0.0000 | 6726.0 | 13.49% | 6227.9 | 12.48% | motif file (matrix) | svg |
| 29 | T G C A C T G A A T G C G T C A A C T G A C T G C G T A C G T A C T A G A G C T | Ets1-distal(ETS)/CD4+-PolII-ChIP-Seq(Barski\_et\_al.)/Homer | 1e-10 | -2.336e+01 | 0.0000 | 2100.0 | 4.21% | 1826.0 | 3.66% | motif file (matrix) | svg |
| 30 | C A T G A G T C G A C T C G T A C G A T G C A T G A C T G C A T C G A T C T A G C A T G T G A C | Mef2b(MADS)/HEK293-Mef2b.V5-ChIP-Seq(GSE67450)/Homer | 1e-9 | -2.239e+01 | 0.0000 | 7821.0 | 15.68% | 7323.1 | 14.68% | motif file (matrix) | svg |
| 31 | G A T C G T A C C G A T A C T G A C T G C G T A C G T A A C G T A C T G G A T C | TEAD(TEA)/Fibroblast-PU.1-ChIP-Seq(Unpublished)/Homer | 1e-9 | -2.197e+01 | 0.0000 | 5343.0 | 10.71% | 4926.1 | 9.87% | motif file (matrix) | svg |
| 32 | T C G A T C G A T A G C G T A C T C A G T A C G C G T A C G T A T C A G A G C T | GABPA(ETS)/Jurkat-GABPa-ChIP-Seq(GSE17954)/Homer | 1e-9 | -2.184e+01 | 0.0000 | 5046.0 | 10.12% | 4641.5 | 9.30% | motif file (matrix) | svg |
| 33 | A G C T C T A G T G A C C G T A A C G T C G A T A G T C A G T C C T G A C A T G | TEAD3(TEA)/HepG2-TEAD3-ChIP-Seq(Encode)/Homer | 1e-9 | -2.166e+01 | 0.0000 | 8826.0 | 17.70% | 8312.1 | 16.66% | motif file (matrix) | svg |
| 34 | A T G C T C G A A G T C A G C T A C G T G T A C A G T C G C T A C T A G C A T G G T C A C T G A T C A G A G T C | Stat3+il21(Stat)/CD4-Stat3-ChIP-Seq(GSE19198)/Homer | 1e-8 | -1.854e+01 | 0.0000 | 5135.0 | 10.30% | 4762.8 | 9.55% | motif file (matrix) | svg |
| 35 | C G T A T A G C T A G C T G C A A C T G C T A G C G T A C G T A T C A G G A C T | EHF(ETS)/LoVo-EHF-ChIP-Seq(GSE49402)/Homer | 1e-7 | -1.699e+01 | 0.0000 | 9238.0 | 18.52% | 8782.0 | 17.60% | motif file (matrix) | svg |
| 36 | C A G T G A C T C G T A G C T A G T A C G A T C G T A C G A C T A G C T A C G T T G A C C G T A C A G T A C G T A T G C | ZNF652/HepG2-ZNF652.Flag-ChIP-Seq(Encode)/Homer | 1e-7 | -1.680e+01 | 0.0000 | 2117.0 | 4.24% | 1886.8 | 3.78% | motif file (matrix) | svg |
| 37 | C A T G G T A C G A C T G C T A C G T A C G T A C G T A G C T A G A C T C T G A T C A G G T A C | Mef2c(MADS)/GM12878-Mef2c-ChIP-Seq(GSE32465)/Homer | 1e-6 | -1.592e+01 | 0.0000 | 4430.0 | 8.88% | 4110.7 | 8.24% | motif file (matrix) | svg |
| 38 | T A C G T C A G A G C T A T G C C G T A A G T C T C A G A C G T A C T G T C G A | USF1(bHLH)/GM12878-Usf1-ChIP-Seq(GSE32465)/Homer | 1e-6 | -1.579e+01 | 0.0000 | 2538.0 | 5.09% | 2294.3 | 4.60% | motif file (matrix) | svg |
| 39 | A T G C G A T C C G A T C T A G A C T G G C T A C G T A A G C T A C T G A G C T | TEAD2(TEA)/Py2T-Tead2-ChIP-Seq(GSE55709)/Homer | 1e-6 | -1.559e+01 | 0.0000 | 3710.0 | 7.44% | 3420.0 | 6.86% | motif file (matrix) | svg |
| 40 | T A C G A T C G G A T C G T C A C T G A G C A T C G A T G C T A T C G A G C T A | Unknown(Homeobox)/Limb-p300-ChIP-Seq/Homer | 1e-6 | -1.540e+01 | 0.0000 | 6611.0 | 13.26% | 6235.2 | 12.50% | motif file (matrix) | svg |
| 41 | G A C T C A G T A G C T C G A T A G T C G A T C A G T C C G T A A T G C T C A G | Rbpj1(?)/Panc1-Rbpj1-ChIP-Seq(GSE47459)/Homer | 1e-6 | -1.451e+01 | 0.0000 | 10274.0 | 20.60% | 9839.1 | 19.72% | motif file (matrix) | svg |
| 42 | C T A G C A T G C A T G T A C G A G T C G C A T A G C T C T A G A C G T A G T C G A C T A C T G A C T G A C T G T C G A | Zfp809(Zf)/ES-Zfp809-ChIP-Seq(GSE70799)/Homer | 1e-6 | -1.414e+01 | 0.0000 | 862.0 | 1.73% | 729.8 | 1.46% | motif file (matrix) | svg |
| 43 | T G C A G C A T A G C T G C A T A G T C A G T C A G T C C T G A A C T G T C G A T C G A C A G T A T C G A G T C G A T C | ZNF143\|STAF(Zf)/CUTLL-ZNF143-ChIP-Seq(GSE29600)/Homer | 1e-6 | -1.399e+01 | 0.0000 | 1458.0 | 2.92% | 1285.9 | 2.58% | motif file (matrix) | svg |
| 44 | T C G A T A G C T G A C C T G A A G T C A C T G G A C T C A T G | c-Myc(bHLH)/LNCAP-cMyc-ChIP-Seq(Unpublished)/Homer | 1e-6 | -1.397e+01 | 0.0000 | 2636.0 | 5.29% | 2404.5 | 4.82% | motif file (matrix) | svg |
| 45 | A G T C C T A G C T A G A G T C G A T C G T A C A G T C C T A G A G T C A G T C A G T C G T A C | Sp2(Zf)/HEK293-Sp2.eGFP-ChIP-Seq(Encode)/Homer | 1e-6 | -1.394e+01 | 0.0000 | 4189.0 | 8.40% | 3900.1 | 7.82% | motif file (matrix) | svg |
| 46 | A C T G G A T C G A C T A C T G A C G T C A T G A C T G A C G T A G C T C G A T | RUNX-AML(Runt)/CD4+-PolII-ChIP-Seq(Barski\_et\_al.)/Homer | 1e-6 | -1.393e+01 | 0.0000 | 5877.0 | 11.78% | 5540.7 | 11.11% | motif file (matrix) | svg |
| 47 | C T G A T C A G G T A C T G C A A G T C C G T A A G T C A C T G A C G T A C T G | MNT(bHLH)/HepG2-MNT-ChIP-Seq(Encode)/Homer | 1e-6 | -1.387e+01 | 0.0000 | 6656.0 | 13.35% | 6301.0 | 12.63% | motif file (matrix) | svg |
| 48 | C T A G G C A T G A T C C G T A A G T C T C A G G A C T C T A G | CLOCK(bHLH)/Liver-Clock-ChIP-Seq(GSE39860)/Homer | 1e-5 | -1.356e+01 | 0.0000 | 3215.0 | 6.45% | 2964.1 | 5.94% | motif file (matrix) | svg |
| 49 | A G T C A C G T A C T G A G C T A C G T A C G T G T C A A G T C | Foxo1(Forkhead)/RAW-Foxo1-ChIP-Seq(Fan\_et\_al.)/Homer | 1e-5 | -1.340e+01 | 0.0000 | 15706.0 | 31.49% | 15228.1 | 30.53% | motif file (matrix) | svg |
| 50 | T C G A T G A C A G T C C G T A A G T C C T A G A C G T A C T G A C T G A G C T A G T C G C A T | Max(bHLH)/K562-Max-ChIP-Seq(GSE31477)/Homer | 1e-5 | -1.302e+01 | 0.0000 | 4033.0 | 8.09% | 3760.7 | 7.54% | motif file (matrix) | svg |
| 51 | G A C T C G A T C T G A G T C A G A C T C G A T T C G A C G T A G C T A G C T A T G A C G T A C C G T A A C T G T G C A C G A T A C T G A C G T | Pitx1:Ebox(Homeobox,bHLH)/Hindlimb-Pitx1-ChIP-Seq(GSE41591)/Homer | 1e-5 | -1.249e+01 | 0.0000 | 2197.0 | 4.41% | 1998.6 | 4.01% | motif file (matrix) | svg |
| 52 | G C T A C T G A T C G A A G T C A G T C C T G A A G T C G T C A C T G A T G C A | RUNX1(Runt)/Jurkat-RUNX1-ChIP-Seq(GSE29180)/Homer | 1e-5 | -1.233e+01 | 0.0000 | 7779.0 | 15.60% | 7425.3 | 14.88% | motif file (matrix) | svg |
| 53 | C G T A T G A C T A G C T G C A A C T G A C T G C G T A C G T A T C A G G A C T | ELF3(ETS)/PDAC-ELF3-ChIP-Seq(GSE64557)/Homer | 1e-5 | -1.199e+01 | 0.0001 | 5229.0 | 10.48% | 4936.9 | 9.90% | motif file (matrix) | svg |
| 54 | A T G C G A T C G A C T A G C T C G A T C G A T G T C A C G A T T C G A A T C G T A G C T A G C | TATA-Box(TBP)/Promoter/Homer | 1e-5 | -1.161e+01 | 0.0001 | 11077.0 | 22.21% | 10685.7 | 21.42% | motif file (matrix) | svg |
| 55 | C A T G A G T C A G C T C G T A C G A T C G A T G C A T G C A T C G A T C T G A C A T G T G A C | Mef2d(MADS)/Retina-Mef2d-ChIP-Seq(GSE61391)/Homer | 1e-4 | -1.151e+01 | 0.0001 | 1887.0 | 3.78% | 1711.4 | 3.43% | motif file (matrix) | svg |
| 56 | T C A G T A G C G A C T C A T G C T G A A T C G G C A T G T A C C G T A A C T G T A G C T G C A | MafK(bZIP)/C2C12-MafK-ChIP-Seq(GSE36030)/Homer | 1e-4 | -1.146e+01 | 0.0001 | 1451.0 | 2.91% | 1297.5 | 2.60% | motif file (matrix) | svg |
| 57 | T C A G A G C T A C G T A C G T G T A C G A T C C G T A C T A G C A T G G T C A C G T A T C G A | STAT4(Stat)/CD4-Stat4-ChIP-Seq(GSE22104)/Homer | 1e-4 | -1.138e+01 | 0.0001 | 8092.0 | 16.22% | 7749.7 | 15.53% | motif file (matrix) | svg |
| 58 | T G C A T C G A T A G C G T A C T C A G C T A G G T C A G C T A T C A G G A C T | ETS(ETS)/Promoter/Homer | 1e-4 | -1.131e+01 | 0.0001 | 1087.0 | 2.18% | 955.1 | 1.91% | motif file (matrix) | svg |
| 59 | C T G A A T G C C G T A A C G T A G T C A G T C A C G T A C T G A T C G G C A T | SPDEF(ETS)/VCaP-SPDEF-ChIP-Seq(SRA014231)/Homer | 1e-4 | -1.008e+01 | 0.0003 | 7163.0 | 14.36% | 6860.5 | 13.75% | motif file (matrix) | svg |
| 60 | C G T A C T A G A C T G A C T G G A C T C T A G C A G T C T A G C A T G G A T C | KLF5(Zf)/LoVo-KLF5-ChIP-Seq(GSE49402)/Homer | 1e-4 | -1.007e+01 | 0.0003 | 3162.0 | 6.34% | 2953.3 | 5.92% | motif file (matrix) | svg |
| 61 | C T A G C A G T T G A C C G T A G A T C T C A G G A C T C A T G | BMAL1(bHLH)/Liver-Bmal1-ChIP-Seq(GSE39860)/Homer | 1e-4 | -1.001e+01 | 0.0003 | 12134.0 | 24.33% | 11764.2 | 23.58% | motif file (matrix) | svg |
| 62 | C A G T A C T G T C A G T G C A G C T A A T G C T C G A A T C G G T C A T G C A | ZNF189(Zf)/HEK293-ZNF189.GFP-ChIP-Seq(GSE58341)/Homer | 1e-4 | -9.911e+00 | 0.0004 | 6624.0 | 13.28% | 6334.3 | 12.70% | motif file (matrix) | svg |
| 63 | G T A C C A G T A C T G A C T G A C T G G A T C A C T G A C G T A C T G A C T G A G T C G A T C | KLF6(Zf)/PDAC-KLF6-ChIP-Seq(GSE64557)/Homer | 1e-4 | -9.891e+00 | 0.0004 | 2889.0 | 5.79% | 2691.2 | 5.39% | motif file (matrix) | svg |
| 64 | T G A C G T C A A G T C G C T A C T A G A G T C C T G A C A T G A C T G C T A G T A C G T C A G | Zic2(Zf)/ESC-Zic2-ChIP-Seq(SRP197560)/Homer | 1e-4 | -9.880e+00 | 0.0004 | 3175.0 | 6.37% | 2968.2 | 5.95% | motif file (matrix) | svg |
| 65 | G C T A C G A T C A T G A T G C A G T C A G T C G A C T T A C G T C G A C T A G A C T G T A G C | AP-2alpha(AP2)/Hela-AP2alpha-ChIP-Seq(GSE31477)/Homer | 1e-4 | -9.804e+00 | 0.0004 | 3305.0 | 6.63% | 3095.4 | 6.20% | motif file (matrix) | svg |
| 66 | A T C G A G T C A G T C G A C T A T G C C T G A C T A G A C T G T A C G G T A C C T G A C G A T | AP-2gamma(AP2)/MCF7-TFAP2C-ChIP-Seq(GSE21234)/Homer | 1e-4 | -9.759e+00 | 0.0004 | 4425.0 | 8.87% | 4185.8 | 8.39% | motif file (matrix) | svg |
| 67 | G A T C T C G A A G T C C G A T C G A T A G T C A T G C A C T G A T C G G A C T | Elk1(ETS)/Hela-Elk1-ChIP-Seq(GSE31477)/Homer | 1e-4 | -9.735e+00 | 0.0004 | 2000.0 | 4.01% | 1837.0 | 3.68% | motif file (matrix) | svg |
| 68 | C G A T T G C A G T A C C G T A A G T C C T A G G A C T C A T G | NPAS(bHLH)/Liver-NPAS-ChIP-Seq(GSE39860)/Homer | 1e-4 | -9.458e+00 | 0.0005 | 11264.0 | 22.58% | 10916.7 | 21.88% | motif file (matrix) | svg |
| 69 | G A C T C G A T T C A G G A T C G A C T A G C T A G C T A G T C G A T C C G T A C T A G C T A G T C G A T C G A C T G A | Bcl6(Zf)/Liver-Bcl6-ChIP-Seq(GSE31578)/Homer | 1e-4 | -9.386e+00 | 0.0005 | 10120.0 | 20.29% | 9787.1 | 19.62% | motif file (matrix) | svg |
| 70 | C G T A C A T G C A T G A C T G C T A G T C G A G C A T C G A T A G C T A G T C G A T C G T A C | NFkB-p65(RHD)/GM12787-p65-ChIP-Seq(GSE19485)/Homer | 1e-4 | -9.246e+00 | 0.0006 | 3182.0 | 6.38% | 2983.6 | 5.98% | motif file (matrix) | svg |
| 71 | T A G C G T A C C G T A C T A G A C T G T G C A C G T A A T G C C G T A A T C G | AR-halfsite(NR)/LNCaP-AR-ChIP-Seq(GSE27824)/Homer | 1e-3 | -9.202e+00 | 0.0006 | 27357.0 | 54.85% | 26950.6 | 54.02% | motif file (matrix) | svg |
| 72 | C G A T C T G A C G T A C A G T A G T C G A T C G A T C A C T G | Pitx1(Homeobox)/Chicken-Pitx1-ChIP-Seq(GSE38910)/Homer | 1e-3 | -9.138e+00 | 0.0007 | 32177.0 | 64.52% | 31788.2 | 63.72% | motif file (matrix) | svg |
| 73 | G C T A T C G A C G T A C T A G A G C T G T C A G T C A C G T A A G T C C G T A | FOXA1(Forkhead)/MCF7-FOXA1-ChIP-Seq(GSE26831)/Homer | 1e-3 | -8.962e+00 | 0.0008 | 10076.0 | 20.20% | 9753.2 | 19.55% | motif file (matrix) | svg |
| 74 | C T G A C T G A C A T G A T C G A G C T A T C G G A C T C A T G C T G A G T C A | Tbr1(T-box)/Cortex-Tbr1-ChIP-Seq(GSE71384)/Homer | 1e-3 | -8.850e+00 | 0.0009 | 12279.0 | 24.62% | 11935.4 | 23.92% | motif file (matrix) | svg |
| 75 | A G T C G A C T C A G T G T A C A G T C A T C G T C A G A C T G G T C A C G T A | Stat3(Stat)/mES-Stat3-ChIP-Seq(GSE11431)/Homer | 1e-3 | -8.840e+00 | 0.0009 | 2993.0 | 6.00% | 2805.8 | 5.62% | motif file (matrix) | svg |
| 76 | C T A G T A G C A G T C G T C A C T G A A C G T C G T A C G T A C G T A G C T A | Hoxd13(Homeobox)/ChickenMSG-Hoxd13.Flag-ChIP-Seq(GSE86088)/Homer | 1e-3 | -8.683e+00 | 0.0010 | 16103.0 | 32.29% | 15734.6 | 31.54% | motif file (matrix) | svg |
| 77 | A T G C C T G A A T C G T A C G A G T C C G A T T C A G C G A T C T A G A G C T G T C A G T C A C G T A A G T C C G T A T A C G C T G A | Fox:Ebox(Forkhead,bHLH)/Panc1-Foxa2-ChIP-Seq(GSE47459)/Homer | 1e-3 | -8.647e+00 | 0.0010 | 9125.0 | 18.30% | 8821.1 | 17.68% | motif file (matrix) | svg |
| 78 | C T G A A G T C C G A T A G C T A T G C G T A C A C G T A T C G C A G T G C A T | Elf4(ETS)/BMDM-Elf4-ChIP-Seq(GSE88699)/Homer | 1e-3 | -8.627e+00 | 0.0010 | 6004.0 | 12.04% | 5749.1 | 11.52% | motif file (matrix) | svg |
| 79 | C A T G T G A C C G T A A G T C T A C G G C A T A C T G G T C A A T G C A G T C | bHLHE41(bHLH)/proB-Bhlhe41-ChIP-Seq(GSE93764)/Homer | 1e-3 | -8.598e+00 | 0.0010 | 6309.0 | 12.65% | 6049.6 | 12.13% | motif file (matrix) | svg |
| 80 | A C T G T G A C G T A C C G T A A G T C T A C G A C G T A C T G G T C A A G T C | NPAS2(bHLH)/Liver-NPAS2-ChIP-Seq(GSE39860)/Homer | 1e-3 | -8.552e+00 | 0.0011 | 6764.0 | 13.56% | 6497.1 | 13.02% | motif file (matrix) | svg |
| 81 | A G T C G A C T A C T G G A T C G T A C C G T A T G A C A G T C C G A T A G C T A C G T A C G T C T A G G A C T C T G A | ZNF7(Zf)/HepG2-ZNF7.Flag-ChIP-Seq(Encode)/Homer | 1e-3 | -8.396e+00 | 0.0012 | 4758.0 | 9.54% | 4533.0 | 9.09% | motif file (matrix) | svg |
| 82 | A G T C G A T C A G T C C G T A A T C G C A G T A G T C G T A C C T G A A C T G T C A G A G C T A G C T A G C T A G C T | PRDM15(Zf)/ESC-Prdm15-ChIP-Seq(GSE73694)/Homer | 1e-3 | -8.353e+00 | 0.0013 | 6701.0 | 13.44% | 6439.4 | 12.91% | motif file (matrix) | svg |
| 83 | A C G T C T A G A G C T A C G T A C G T C T G A A G T C G A C T A G C T C G T A | FOXM1(Forkhead)/MCF7-FOXM1-ChIP-Seq(GSE72977)/Homer | 1e-3 | -8.277e+00 | 0.0013 | 10239.0 | 20.53% | 9930.5 | 19.91% | motif file (matrix) | svg |
| 84 | A T G C C T G A G A C T A C G T A C G T G T A C G A T C C G A T C T A G C A T G C G T A C G T A C T G A G A C T | STAT1(Stat)/HelaS3-STAT1-ChIP-Seq(GSE12782)/Homer | 1e-3 | -8.237e+00 | 0.0014 | 2286.0 | 4.58% | 2128.3 | 4.27% | motif file (matrix) | svg |
| 85 | G C A T G C A T C T G A A C G T C T G A A C G T C G T A C G T A C G T A A G T C G T C A G T C A | Foxf1(Forkhead)/Lung-Foxf1-ChIP-Seq(GSE77951)/Homer | 1e-3 | -8.197e+00 | 0.0014 | 8980.0 | 18.01% | 8688.4 | 17.42% | motif file (matrix) | svg |
| 86 | A G T C C G A T C T G A C G T A A C G T C A G T T C A G T G A C | Isl1(Homeobox)/Neuron-Isl1-ChIP-Seq(GSE31456)/Homer | 1e-3 | -8.187e+00 | 0.0014 | 17151.0 | 34.39% | 16790.4 | 33.66% | motif file (matrix) | svg |
| 87 | C G T A A C G T A C T G G T A C C G T A A C G T C G T A C G T A A C G T A C T G A G T C C G T A A C G T C T G A G C A T | OCT:OCT-short(POU,Homeobox)/NPC-OCT6-ChIP-Seq(GSE43916)/Homer | 1e-3 | -8.018e+00 | 0.0017 | 7728.0 | 15.50% | 7457.6 | 14.95% | motif file (matrix) | svg |
| 88 | C A T G A C G T A G T C G A T C G A T C G A T C G C T A C T A G C T A G C T A G T C A G T C G A | EBF1(EBF)/Near-E2A-ChIP-Seq(GSE21512)/Homer | 1e-3 | -7.916e+00 | 0.0018 | 5068.0 | 10.16% | 4844.8 | 9.71% | motif file (matrix) | svg |
| 89 | A G C T A C G T A C T G A T G C A G T C C G T A C T G A T A C G | NF1-halfsite(CTF)/LNCaP-NF1-ChIP-Seq(Unpublished)/Homer | 1e-3 | -7.881e+00 | 0.0019 | 13567.0 | 27.20% | 13237.7 | 26.54% | motif file (matrix) | svg |
| 90 | A C T G C A G T A C G T C G T A C G T A A C G T A C T G C T G A | Nkx6.1(Homeobox)/Islet-Nkx6.1-ChIP-Seq(GSE40975)/Homer | 1e-3 | -7.875e+00 | 0.0019 | 24475.0 | 49.07% | 24105.1 | 48.32% | motif file (matrix) | svg |
| 91 | T G A C C T G A C T A G C T G A C G T A A G T C C T G A A C G T G C A T T A G C G C A T A T C G G A C T G A C T G A T C | GRE(NR),IR3/RAW264.7-GRE-ChIP-Seq(Unpublished)/Homer | 1e-3 | -7.824e+00 | 0.0019 | 1991.0 | 3.99% | 1848.8 | 3.71% | motif file (matrix) | svg |
| 92 | C T G A C T A G T G C A C T G A T C G A A G C T C A T G T C G A A G T C G A C T A C G T A G T C G A T C G A T C G A C T | ZNF528(Zf)/HEK293-ZNF528.GFP-ChIP-Seq(GSE58341)/Homer | 1e-3 | -7.776e+00 | 0.0020 | 37.0 | 0.07% | 20.1 | 0.04% | motif file (matrix) | svg |
| 93 | C G A T C T A G A C G T A C G T A C G T C G T A A G C T C G A T A G C T C G T A C T A G T A G C | FoxD3(forkhead)/ZebrafishEmbryo-Foxd3.biotin-ChIP-seq(GSE106676)/Homer | 1e-3 | -7.684e+00 | 0.0022 | 8728.0 | 17.50% | 8451.3 | 16.94% | motif file (matrix) | svg |
| 94 | C A T G T A C G T A G C G A T C G A T C A T G C G T A C G A C T T C A G A T G C C G A T A T C G C A G T A C T G G T A C | Zic3(Zf)/mES-Zic3-ChIP-Seq(GSE37889)/Homer | 1e-3 | -7.596e+00 | 0.0024 | 4096.0 | 8.21% | 3898.8 | 7.82% | motif file (matrix) | svg |
| 95 | G C T A G A C T G A C T T G C A C G T A A G T C C G T A T A G C G A T C G A C T | Eomes(T-box)/H9-Eomes-ChIP-Seq(GSE26097)/Homer | 1e-3 | -7.465e+00 | 0.0027 | 18334.0 | 36.76% | 17989.6 | 36.06% | motif file (matrix) | svg |
| 96 | C G A T A C G T A C G T A C G T C G T A A G C T C A G T C T A G A T C G A C T G | HOXB13(Homeobox)/ProstateTumor-HOXB13-ChIP-Seq(GSE56288)/Homer | 1e-3 | -7.457e+00 | 0.0027 | 10251.0 | 20.55% | 9962.9 | 19.97% | motif file (matrix) | svg |
| 97 | G A C T C T A G C T A G C T A G A C T G T C G A C T G A C T A G C T A G C T A G G T A C G T C A | ZNF467(Zf)/HEK293-ZNF467.GFP-ChIP-Seq(GSE58341)/Homer | 1e-3 | -7.450e+00 | 0.0027 | 3937.0 | 7.89% | 3745.6 | 7.51% | motif file (matrix) | svg |
| 98 | C T G A A G C T A C G T A C G T A G T C G A C T G A C T C T G A C T G A C T A G C G T A C G T A | STAT6(Stat)/CD4-Stat6-ChIP-Seq(GSE22104)/Homer | 1e-3 | -7.396e+00 | 0.0028 | 5098.0 | 10.22% | 4883.5 | 9.79% | motif file (matrix) | svg |
| 99 | C A G T C G A T G C A T G C A T G T C A A G C T C A T G C T A G A T G C G T A C | Hoxa11(Homeobox)/ChickenMSG-Hoxa11.Flag-ChIP-Seq(GSE86088)/Homer | 1e-3 | -7.345e+00 | 0.0029 | 22535.0 | 45.18% | 22183.1 | 44.47% | motif file (matrix) | svg |
| 100 | A G C T T G A C C G A T C G A T C T A G A C G T C A G T C A G T G C T A A G T C | FOXK1(Forkhead)/HEK293-FOXK1-ChIP-Seq(GSE51673)/Homer | 1e-3 | -7.339e+00 | 0.0029 | 9511.0 | 19.07% | 9233.5 | 18.51% | motif file (matrix) | svg |
| 101 | T C A G C T A G C T A G C T A G T C A G T C G A C T G A C G A T A G T C G A T C A G T C T G A C | NFkB-p50,p52(RHD)/Monocyte-p50-ChIP-Chip(Schreiber\_et\_al.)/Homer | 1e-3 | -7.268e+00 | 0.0030 | 472.0 | 0.95% | 406.8 | 0.82% | motif file (matrix) | svg |
| 102 | T A C G C T G A T C G A C G A T C T A G C T A G T C G A C T G A T C G A T C G A C G T A T C G A G C A T C A T G C G T A T A C G G C A T T G A C C G T A A G C T | NFAT:AP1(RHD,bZIP)/Jurkat-NFATC1-ChIP-Seq(Jolma\_et\_al.)/Homer | 1e-3 | -7.186e+00 | 0.0033 | 1479.0 | 2.97% | 1362.4 | 2.73% | motif file (matrix) | svg |
| 103 | G C T A T C G A C G T A C T A G A G C T G T C A G T C A C G T A A G T C C G T A | FOXA1(Forkhead)/LNCAP-FOXA1-ChIP-Seq(GSE27824)/Homer | 1e-3 | -7.167e+00 | 0.0033 | 12015.0 | 24.09% | 11717.0 | 23.49% | motif file (matrix) | svg |
| 104 | A T G C T C G A T A C G A C G T A T G C A G T C A C G T A G T C A G T C G A T C | Znf263(Zf)/K562-Znf263-ChIP-Seq(GSE31477)/Homer | 1e-3 | -6.959e+00 | 0.0040 | 11984.0 | 24.03% | 11692.0 | 23.44% | motif file (matrix) | svg |
| 105 | T C A G A G C T A T G C C G T A A G C T T C A G C A G T A C T G C T G A A G T C | MITF(bHLH)/MastCells-MITF-ChIP-Seq(GSE48085)/Homer | 1e-3 | -6.952e+00 | 0.0040 | 5072.0 | 10.17% | 4866.9 | 9.76% | motif file (matrix) | svg |
| 106 | C T G A C A G T C T G A A G T C C T A G G A C T A T C G G T A C | HIF-1b(HLH)/T47D-HIF1b-ChIP-Seq(GSE59937)/Homer | 1e-3 | -6.931e+00 | 0.0041 | 8568.0 | 17.18% | 8311.0 | 16.66% | motif file (matrix) | svg |
| 107 | A G T C G A C T C A G T A C T G C T A G T G A C G C T A A T G C G C A T A T C G C G A T A C T G G A T C G T A C G T C A C T G A | NF1(CTF)/LNCAP-NF1-ChIP-Seq(Unpublished)/Homer | 1e-2 | -6.890e+00 | 0.0042 | 1966.0 | 3.94% | 1835.8 | 3.68% | motif file (matrix) | svg |
| 108 | C G T A C T A G T C A G T C A G A G T C A T G C A G T C G C A T A G C T A C G T A T C G C G A T | Sox9(HMG)/Limb-SOX9-ChIP-Seq(GSE73225)/Homer | 1e-2 | -6.790e+00 | 0.0046 | 6358.0 | 12.75% | 6134.4 | 12.30% | motif file (matrix) | svg |
| 109 | T A G C G A T C G T A C C T G A G C A T C T G A C G T A T G C A C G T A G A T C | Hoxa13(Homeobox)/ChickenMSG-Hoxa13.Flag-ChIP-Seq(GSE86088)/Homer | 1e-2 | -6.643e+00 | 0.0053 | 23666.0 | 47.45% | 23336.2 | 46.78% | motif file (matrix) | svg |
| 110 | A G T C C T G A A T C G A G C T A G C T G A C T A G T C G C T A A C G T C G A T G C A T C G A T A T C G C G T A T A G C G C A T A T G C C G T A | bZIP:IRF(bZIP,IRF)/Th17-BatF-ChIP-Seq(GSE39756)/Homer | 1e-2 | -6.639e+00 | 0.0053 | 3934.0 | 7.89% | 3756.9 | 7.53% | motif file (matrix) | svg |
| 111 | A T G C A G T C G T A C A G C T T C G A C T A G G A T C C T G A G T C A A G T C G C T A T C A G | Rfx5(HTH)/GM12878-Rfx5-ChIP-Seq(GSE31477)/Homer | 1e-2 | -6.627e+00 | 0.0053 | 1975.0 | 3.96% | 1848.0 | 3.70% | motif file (matrix) | svg |
| 112 | G C T A A G T C T A C G T G C A A T C G T C A G G C T A T C G A T C A G A G C T | ELF5(ETS)/T47D-ELF5-ChIP-Seq(GSE30407)/Homer | 1e-2 | -6.601e+00 | 0.0053 | 4956.0 | 9.94% | 4759.3 | 9.54% | motif file (matrix) | svg |
| 113 | G T C A T G C A G C T A A G T C C G T A A C T G T G A C G C A T T C A G C A G T | Ap4(bHLH)/AML-Tfap4-ChIP-Seq(GSE45738)/Homer | 1e-2 | -6.587e+00 | 0.0054 | 11685.0 | 23.43% | 11406.2 | 22.86% | motif file (matrix) | svg |
| 114 | A C G T T A C G G A T C A C T G A C G T C T A G A C T G A C T G G A T C C T A G C A T G C T A G | Egr2(Zf)/Thymocytes-Egr2-ChIP-Seq(GSE34254)/Homer | 1e-2 | -6.559e+00 | 0.0055 | 376.0 | 0.75% | 321.3 | 0.64% | motif file (matrix) | svg |
| 115 | G A T C C T G A A G T C C G A T C G A T G A T C A G T C A C T G A T C G A G C T | Elk4(ETS)/Hela-Elk4-ChIP-Seq(GSE31477)/Homer | 1e-2 | -6.334e+00 | 0.0068 | 2033.0 | 4.08% | 1907.8 | 3.82% | motif file (matrix) | svg |
| 116 | T C G A T G A C G T A C C G T A C A G T T G A C A C G T A C T G A G C T A G C T | NeuroG2(bHLH)/Fibroblast-NeuroG2-ChIP-Seq(GSE75910)/Homer | 1e-2 | -6.322e+00 | 0.0068 | 13114.0 | 26.29% | 12832.9 | 25.72% | motif file (matrix) | svg |
| 117 | T C G A T G C A A C T G G T C A A G T C A G T C A G T C A G T C A G C T T G A C | LRF(Zf)/Erythroblasts-ZBTB7A-ChIP-Seq(GSE74977)/Homer | 1e-2 | -6.303e+00 | 0.0069 | 4841.0 | 9.71% | 4653.0 | 9.33% | motif file (matrix) | svg |
| 118 | A T G C A G T C G C T A C G A T C G T A G C A T G C T A C G A T C T A G C A T G T G A C G T C A | CArG(MADS)/PUER-Srf-ChIP-Seq(Sullivan\_et\_al.)/Homer | 1e-2 | -6.249e+00 | 0.0072 | 2105.0 | 4.22% | 1978.5 | 3.97% | motif file (matrix) | svg |
| 119 | C G T A C A T G A G T C G A C T T G C A C G T A A C G T A C G T C T G A T C A G | Lhx1(Homeobox)/EmbryoCarcinoma-Lhx1-ChIP-Seq(GSE70957)/Homer | 1e-2 | -6.173e+00 | 0.0077 | 11944.0 | 23.95% | 11675.8 | 23.40% | motif file (matrix) | svg |
| 120 | T C A G A G C T G T A C C G T A A C G T C G T A C G T A C G T A G C T A G A C T | Cdx2(Homeobox)/mES-Cdx2-ChIP-Seq(GSE14586)/Homer | 1e-2 | -6.127e+00 | 0.0080 | 6941.0 | 13.92% | 6724.7 | 13.48% | motif file (matrix) | svg |
| 121 | A G T C T A G C G A C T A C G T C T A G A C G T A C G T A C G T C T G A A G T C G C T A G A C T C G T A C T A G A C T G | Foxa3(Forkhead)/Liver-Foxa3-ChIP-Seq(GSE77670)/Homer | 1e-2 | -6.119e+00 | 0.0080 | 2855.0 | 5.72% | 2710.6 | 5.43% | motif file (matrix) | svg |
| 122 | C A T G G T A C C G T A A G T C C T A G A C G T A C T G G T A C A G T C A G C T | bHLHE40(bHLH)/HepG2-BHLHE40-ChIP-Seq(GSE31477)/Homer | 1e-2 | -6.029e+00 | 0.0087 | 1869.0 | 3.75% | 1752.1 | 3.51% | motif file (matrix) | svg |
| 123 | T A C G C T G A C T A G T C G A C G T A A G T C C T G A A T C G G C A T T A G C G A C T A C T G A C G T A G C T A G T C G A C T | GRE(NR),IR3/A549-GR-ChIP-Seq(GSE32465)/Homer | 1e-2 | -6.023e+00 | 0.0087 | 1126.0 | 2.26% | 1035.2 | 2.08% | motif file (matrix) | svg |
| 124 | G A C T C A G T G A T C G A T C A C G T G A T C C T G A T A C G C G T A G T C A | STAT6(Stat)/Macrophage-Stat6-ChIP-Seq(GSE38377)/Homer | 1e-2 | -5.914e+00 | 0.0096 | 5084.0 | 10.19% | 4899.4 | 9.82% | motif file (matrix) | svg |
| 125 | T A C G T A G C G C T A C G A T C T A G A C G T C A G T C A G T G C T A A G T C G T C A G C A T | FOXK2(Forkhead)/U2OS-FOXK2-ChIP-Seq(E-MTAB-2204)/Homer | 1e-2 | -5.782e+00 | 0.0108 | 5659.0 | 11.35% | 5468.2 | 10.96% | motif file (matrix) | svg |
| 126 | T C G A G C A T A C T G C T G A A G T C T C A G G A C T G T A C C G T A A G C T A G T C G A T C | c-Jun-CRE(bZIP)/K562-cJun-ChIP-Seq(GSE31477)/Homer | 1e-2 | -5.775e+00 | 0.0108 | 1781.0 | 3.57% | 1670.9 | 3.35% | motif file (matrix) | svg |
| 127 | T C A G T A C G G A T C G T A C T C G A C G A T C T G A C G T A G C T A C G T A | Hoxd11(Homeobox)/ChickenMSG-Hoxd11.Flag-ChIP-Seq(GSE86088)/Homer | 1e-2 | -5.729e+00 | 0.0113 | 22627.0 | 45.37% | 22330.4 | 44.76% | motif file (matrix) | svg |
| 128 | T C A G G A C T C A G T C T G A A G C T C T A G G A C T T G C A C T G A A G T C | HLF(bZIP)/HSC-HLF.Flag-ChIP-Seq(GSE69817)/Homer | 1e-2 | -5.681e+00 | 0.0117 | 8237.0 | 16.52% | 8016.2 | 16.07% | motif file (matrix) | svg |
| 129 | T G A C A G T C C G T A A C T G G T A C A C G T A C T G A C G T G A C T G A T C | Twist2(bHLH)/Myoblast-Twist2.Ty1-ChIP-Seq(GSE127998)/Homer | 1e-2 | -5.679e+00 | 0.0117 | 15072.0 | 30.22% | 14800.0 | 29.67% | motif file (matrix) | svg |
| 130 | T G C A T C G A T C G A A C G T C A T G C G T A A G T C T C A G A C G T G T A C C G T A A G C T | CREB5(bZIP)/LNCaP-CREB5.V5-ChIP-Seq(GSE137775)/Homer | 1e-2 | -5.555e+00 | 0.0131 | 2693.0 | 5.40% | 2561.1 | 5.13% | motif file (matrix) | svg |
| 131 | T A C G T C A G G A T C G T A C T C G A G A C T G C T A G C T A G C T A C G T A G A T C G T C A | CDX4(Homeobox)/ZebrafishEmbryos-Cdx4.Myc-ChIP-Seq(GSE48254)/Homer | 1e-2 | -5.530e+00 | 0.0133 | 9039.0 | 18.12% | 8814.4 | 17.67% | motif file (matrix) | svg |
| 132 | C G T A G C T A C G A T C T A G A C G T G T C A C G T A C G T A A G T C C G T A T G C A T A C G | FoxL2(Forkhead)/Ovary-FoxL2-ChIP-Seq(GSE60858)/Homer | 1e-2 | -5.525e+00 | 0.0133 | 8073.0 | 16.19% | 7858.9 | 15.75% | motif file (matrix) | svg |
| 133 | A T G C G A C T A G C T A G C T A G T C G C T A C A G T C G A T G C T A A C G T A C T G G C T A T A G C G C A T T G A C | IRF:BATF(IRF:bZIP)/pDC-Irf8-ChIP-Seq(GSE66899)/Homer | 1e-2 | -5.391e+00 | 0.0151 | 1115.0 | 2.24% | 1031.7 | 2.07% | motif file (matrix) | svg |
| 134 | T G A C G C T A T C G A T G C A A G T C A G T C C G T A A G T C C G T A C T G A G C T A G T A C | RUNX2(Runt)/PCa-RUNX2-ChIP-Seq(GSE33889)/Homer | 1e-2 | -5.388e+00 | 0.0151 | 6418.0 | 12.87% | 6226.2 | 12.48% | motif file (matrix) | svg |
| 135 | T A G C G C T A T C G A C T G A A G T C A G T C C T G A A G T C C G T A C T A G | RUNX(Runt)/HPC7-Runx1-ChIP-Seq(GSE22178)/Homer | 1e-2 | -5.358e+00 | 0.0154 | 4971.0 | 9.97% | 4800.9 | 9.62% | motif file (matrix) | svg |
| 136 | A G T C A G T C C G A T A C G T A C G T A C T G A C G T A G C T A G T C A G T C | Sox4(HMG)/proB-Sox4-ChIP-Seq(GSE50066)/Homer | 1e-2 | -5.339e+00 | 0.0155 | 6677.0 | 13.39% | 6483.3 | 13.00% | motif file (matrix) | svg |
| 137 | C T A G A T C G G A C T A G T C A G T C A G T C G A C T C T G A A C T G C T A G A C T G C T G A | EBF(EBF)/proBcell-EBF-ChIP-Seq(GSE21978)/Homer | 1e-2 | -5.151e+00 | 0.0186 | 998.0 | 2.00% | 921.0 | 1.85% | motif file (matrix) | svg |
| 138 | C G A T C T A G T C A G C A G T C G T A A G T C G C T A A C G T G A C T A T G C A G T C G C T A | PRDM10(Zf)/HEK293-PRDM10.eGFP-ChIP-Seq(Encode)/Homer | 1e-2 | -4.950e+00 | 0.0226 | 3628.0 | 7.27% | 3488.3 | 6.99% | motif file (matrix) | svg |
| 139 | T G A C C T A G T C A G G T C A C G T A T C A G C G A T T C A G T C G A T G C A C T G A T A G C | PU.1-IRF(ETS:IRF)/Bcell-PU.1-ChIP-Seq(GSE21512)/Homer | 1e-2 | -4.884e+00 | 0.0240 | 10273.0 | 20.60% | 10057.7 | 20.16% | motif file (matrix) | svg |
| 140 | C T G A C A T G A C T G A C G T A T G C C G T A C A T G T A C G A T G C G C T A T A C G C T G A C T A G A C T G A C G T A T G C C G T A T A G C | RAR:RXR(NR),DR5/ES-RAR-ChIP-Seq(GSE56893)/Homer | 1e-2 | -4.876e+00 | 0.0240 | 112.0 | 0.22% | 88.7 | 0.18% | motif file (matrix) | svg |
| 141 | C G T A C T A G C A T G A G C T A C T G C G A T A T C G C G T A G T C A G T C A | Tbet(T-box)/CD8-Tbet-ChIP-Seq(GSE33802)/Homer | 1e-2 | -4.855e+00 | 0.0243 | 8604.0 | 17.25% | 8403.6 | 16.85% | motif file (matrix) | svg |
| 142 | A G T C A T C G G C A T C T A G A C T G T A C G C G A T T C A G C A T G A G C T T A G C G A T C | GLI3(Zf)/Limb-GLI3-ChIP-Chip(GSE11077)/Homer | 1e-2 | -4.816e+00 | 0.0251 | 448.0 | 0.90% | 399.2 | 0.80% | motif file (matrix) | svg |
